# Impact of genetic variation in the human leptin gene promoter on metabolic dysfunction-associated steatotic liver disease risk

**DOI:** 10.20945/2359-4292-2024-0458

**Published:** 2025-09-28

**Authors:** Fatemeh Ghasemi, Mitra Rostami, Zahra Ourang, Atefeh Dehghanitafti, Nikta Zafarjafarzadeh, Amirhesam Mashaollahi, Kosar Babaeian Roshani, Aidin Mahban, Mobina Hosseini, Touraj Mahmoudi, Gholamreza Rezamand, Asadollah Asadi, Hossein Nobakht, Reza Dabiri, Hamid Farahani, Seidamir Pasha Tabaeian

**Affiliations:** 1 Gastroenterology and Liver Diseases Research Center, Research Institute for Gastroenterology and Liver Diseases, Shahid Beheshti University of Medical Sciences, Tehran, Iran; 2 Department of Business Management, Science and Research Branch, Islamic Azad University, Tehran, Iran; 3 Department of Internal Medicine, School of Medicine, Iran University of Medical Sciences, Tehran, Iran; 4 Colorectal Research Center, Iran University of Medical Sciences, Tehran, Iran; 5 Department of Biology, Faculty of Science, University of Mohaghegh Ardabili, Ardabil, Iran; 6 Internal Medicine Department, Semnan University of Medical Sciences, Semnan, Iran; 7 Department of Physiology and Pharmacology, School of Medicine, Qom University of Medical Sciences, Qom, Iran

**Keywords:** -2548G>A, leptin, MASLD, rs7799039, variant

## Abstract

**Objective:**

Metabolic dysfunction-associated steatotic liver disease (MASLD), a worldwide public health challenge with a prevalence of around 25%, is strongly related to obesity and insulin resistance. The present study investigated the possible association between MASLD and the leptin gene (*LEP*) -2548G>A (rs7799039) polymorphism.

**Subjects and methods:**

A total of 250 subjects (125 biopsy-proven MASLD patients and 125 controls) were genotyped for the -2548G>A promoter variant using the PCR-RFLP technique.

**Results:**

There was no deviation from Hardy-Weinberg equilibrium for *LEP* -2548G>A polymorphism in both groups (P > 0.05). A significant association between this gene variant and MASLD was found. The* LEP* -2548G>A “GG” genotype compared with ‘‘AA+AG’’ genotype was underrepresented in the MASLD patients than controls, even after adjustment for confounding factors (*P* = 0.016; OR = 0.42, 95% CI = 0.40-0.83).

**Conclusion:**

For the first time, our findings demonstrated that the “GG” genotype of *LEP* -2548G>A gene variant can be a potential protective factor for MASLD. Further studies in other populations, however, are required to support this finding.

## INTRODUCTION

Metabolic dysfunction-associated steatotic liver disease (MASLD), formerly known as nonalcoholic fatty liver disease (NAFLD), is a global public health challenge characterized by abnormal lipid accumulation in the liver without excessive alcohol consumption or other causes of lipid buildup. MASLD begins with steatosis, progresses to steatohepatitis and can lead to fibrosis and cirrhosis. The prevalence of MASLD, currently around 25%, has increased in recent decades due to rising rates of obesity and type 2 diabetes (T2D) (^1^,^2^).

The complex mechanism underlying the development and progression of MASLD are still not fully understood, but there is growing evidence linking MASLD to obesity (^3^,^4^) and insulin resistance (IR) (^5^,^6^). IR, a key and very important feature of MASLD, is strongly associated with hepatic steatosis and liver fibrosis (^7^,^8^). Individuals with MASLD are also at higher risk for T2D (^4^), hyperinsulinemia (^9^), dyslipidemia (^4^), and high blood pressure (^4^).

Adipokines are a family of polypeptides originating from adipose tissue. Leptin as an adipokine is a multifunctional protein that may play a role in the pathogenesis of MASLD by contributing to various metabolic disorders associated with the disease, including obesity, IR, inflammation, hypertension, dyslipidemia, and T2D (^10^). Leptin, a 16-kDa single chain polypeptide hormone, is the primary adipokine that reflects the amount of fat stored in adipocytes. It regulates satiety, appetite, body fat, lipid metabolism, insulin sensitivity, insulin secretion, and glucose tolerance. Leptin reduces food intake, stimulates energy expenditure (^11^), and is positively associated with insulin levels (^12^), fasting blood glucose (^5^), obesity (^5^,^13^), and IR (^5^,^12^). The leptin to adiponectin ratio also has positive links with BMI and hepatic steatosis, and it is higher in MASLD too (^14^).

Studies have shown conflicting results regarding circulating leptin levels in MASLD patients, with some indicating higher levels compared to control (^5^,^12^-^15^) and others indicating no significant difference (^16^,^17^). Variants in the *LEP* gene, which encodes the leptin protein, have been associated with circulating leptin levels (^18^-^21^), glucose levels (^21^), IR (^20^,^21^), triglycerides (^22^), total cholesterol (^18^), dyslipidemia (^23^), T2DM (^24^), and hypertension (^25^,^26^). This study aimed to investigate the potential relationship between the *LEP* gene -2548G>A (rs7799039) variant and MASLD risk due to its location in the *LEP* gene promoter, a minor allele frequency (MAF) of at least 20%, and previous use in the literature.

## SUBJECTS AND METHODS

### Study population

One hundred and twenty-five patients diagnosed with biopsy-proven MASLD and an equal number of controls were selected as research subjects (**Table 1**). The diagnosis of MASLD was based on a) the ultrasonic diagnostic criteria for fatty liver b) elevated liver enzymes such as aspartate aminotransferase (AST), alanine aminotransferase (ALT), and gamma glutamyl transferase (GGT) c) absence of other causes of hepatic fat accumulation and liver disease, e.g. viral hepatitis, alpha-1 antitrypsin deficiency, Wilson’s disease, significant alcohol consumption (>20 g/day or 140 g/week in men and 10 g/day or 70 g/week in women), and medications that cause steatosis, and d) evidence of MASLD by liver biopsy according to Brunt’s criteria (steatosis grades: 0-3; necroinflammation grades: 0-3; fibrosis stages: 0-4).

**Table 1:** Demographic, anthropometric, biochemical, and clinical data of MASLD and control groups^a^

Characteristic	MASLD	Controls	*P*-value
No. of subjects	125	125	
Gender			
Males/Females	79 (63.2) / 46 (36.8)	73 (58.4) / 52 (41.6)	0.342
Age (years)	35.4 (7.1)	33.6 (6.2)	
Range	30-65	32-58	0.178
BMI (kg/m^2^)	26.8 (3.6)	25.1 (3.2)	0.085
Current or former smoker	23 (18.4)	19 (15.2)	0.437
SBP (mmHg)	128.2 (17.0)	115.6 (14.1)	<0.001
DBP (mmHg)	80.4 (10.7)	71.9 (8.5)	<0.001
ALT (IU/L)	67.6 (35.2)	18.3 (8.4)	<0.001
AST (IU/L)	36.9 (18.5)	18.1 (6.7)	<0.001
GGT (IU/L)	53.3 (32.9)	20.3 (7.1)	<0.001
T2D	23 (18.4)		
Hypertension	26 (20.8)		
Steatosis			
Grade 0 and 1	22 (17.6)		
Grade 2 and 3	103 (82.4)		
Necroinflammation			
Grade 0 and 1	72 (57.6)		
Grade 2 and 3	53 (42.4)		
Fibrosis			
Stage 0 and 1	98 (78.4)		
Stage 2, 3, and 4	27 (21.6)		

ALT: alanine aminotransferase; AST: aspartate aminotransferase; BMI: body mass index; DBP: diastolic blood pressure; GGT: gamma glutamyl transferase; MASLD: metabolic dysfunction-associated steatotic liver disease; SBP: systolic blood pressure; T2D: type 2 diabetes.

a Data are mean (SD) or number (%).

One hundred and twenty-five participants with ultrasonographically normal livers were recruited from the Institute personnel and students (Research Institute for Gastroenterology and Liver Diseases, Shahid Beheshti University of Medical Sciences) as controls. The controls had no history of elevated AST, ALT, and GGT, viral hepatitis (confirmed by blood test), liver steatosis (confirmed by abdominal ultrasonography), alcoholism or regular medication usage. All subjects voluntarily participated in the study after providing informed consent, and a self-report questionnaire was used to collect participant information (^27^,^28^). This research was conducted in accordance with the Declaration of Helsinki and its amendments as a statement of ethical principles. Approval was obtained from the Institute’s Ethics Committee (the ethics committee number: 1430).

### SNP genotyping

The DNA was extracted from 5 mL EDTA-anticoagulated venous blood by phenol-chloroform extraction and ethanol precipitation, then stored at -20 ºC. For the detection of the genotypes of the *LEP* gene -2548G>A variant, the polymerase chain reaction-restriction fragment length polymorphism method was used. The PCR products were digested using the restriction enzyme HhaI (Fermentas, Leon-Rot, Germany) in a water bath at 37 ºC (**Table 2**). The digested PCR products (RFLP fragments) were separated on 3.0% agarose gels, stained with 0.5 µg/mL ethidium bromide and visualized with a UV transilluminator (^29^). *LEP* -2548G>A genotyping was determined by the digestion pattern of the HhaI enzyme. The genotyping process was verified by re-genotyping approximately 20% of participants, with 100% reproducibility.

**Table 2:** The studied variant in leptin gene (*LEP*)

Gene (polymorphism) Location (Base change)	Primer sequence (forward and reverse)	PCR annealing temperature	PCR product size (bp)	Restriction Enzyme	RFLP products size (bp)
*LEP* (rs7799039) Promoter (G/A)	5’-TTTCCTGTAATTTTCCCGTGAG-3’ 5’-AAAGCAAAGACAGGCATAAAAA-3’	61 ºC	242	HhaI	A: 242 G: 181+61

### Statistical analyses

T-tests were used to compare continuous variables. Chi-square tests were used to compare categorical clinical variables and allele frequencies between case and control groups, as well as to verify genotype distributions with the Hardy-Weinberg equilibrium (HWE). Logistic regression was used to evaluate the relationship between MASLD and genotype frequencies, as well as to adjust the results with age, body mass index, sex, smoking history, systolic blood pressure, diastolic blood pressure, hypertension, and type 2 diabetes. Odds ratios and 95% confidence intervals were used to measure the association between the -2548G>A polymorphism of the *LEP* gene and MASLD. A statistically significant difference was defined as a p-value less than 0.05 (SPSS, version 25.0, Chicago, IL, USA).

## RESULTS

### Clinicopathological and biochemical analysis

The clinical and biochemical characteristics of the individuals are presented in **Table 1**. There were statistically significant differences between the patients with MASLD and the controls in terms of systolic blood pressure (SBP), diastolic blood pressure (DBP), and circulating levels of AST, ALT, and GGT; all of which were higher in the patients (*P* < 0.001).

### *LEP* gene polymorphism analysis

**Table 3** shows the comparison between MASLD and control groups regarding *LEP* -2548G>A gene polymorphism. The SNP genotype frequencies in both groups, cases and controls, were consistent with the HWE (*P* > 0.05), indicating that the participants in this study could represent the general population. We found a significant association between this gene variant and MASLD. The *LEP* -2548G>A “GG” genotype compared with the ‘‘AA+AG’’ genotype was less common in the biopsy-proven MASLD patients than in controls, even after adjusting for confounding factors (*P* = 0.016; OR = 0.42, 95% CI = 0.40-0.83). However, no significant difference was detected between these two groups regarding the *LEP* -2548G>A allele frequencies (*P* = 0.320).

**Table 3:** Distribution of leptin gene (*LEP*) rs7799039 polymorphism in the patients with metabolic dysfunction-associated steatotic liver and in the controls^a^

Gene (variant)	Controls (n = 125)	Patients (n = 125)	OR (95% CI) P^b^
*LEP* (rs7799039)			
Genotype-wise comparison			
AA	40 (32.0)	44 (35.2)	1.0 (reference)
AG	55 (44.0)	58 (46.4)	1.12 (0.48-2.44) 0.879
GG	30 (24.0)	23 (18.4)	0.71 (0.69-1.18) 0.166
AG and GG	85 (68.0)	81 (64.8)	0.86 (0.49-1.53) 0.604
GG versus others	30 (24.0)	23 (18.4)	0.42 (0.40-0.83) 0.016
Allele-wise comparison			
A	135 (54.0)	146 (58.4)	1.0 (reference)
G	115 (46.0)	104 (41.6)	0.81 (0.53-1.29) 0.320

a Variables presented as number (%).

b Adjusted for age, body mass index, sex, smoking status, systolic blood pressure, diastolic blood pressure, hypertension, and type 2 diabetes in genotype-wise comparisons.

## DISCUSSION

This research was conducted to investigate the possible role of the *LEP* gene -2548G>A polymorphism in susceptibility to MASLD. Our findings suggest that the *LEP* -2548G>A “GG” genotype may be a potential protective factor for biopsy-proven MASLD. This finding aligns with previous reports indicating a negative association between the “GG” genotype and leptin secretion, IR, and obesity.

MASLD is a complex metabolic condition that is rapidly becoming a significant global health issue. The intricate interplay between genetic and environmental factors contributes to the development and progression of MASLD. Genome-wide association studies have identified numerous variants linked to the susceptibility of complex diseases like MASLD (^30^). However, inconsistencies in genetic association studies are common due to variations in environmental factors, differences in disease definition, racial disparities in genetic makeup, and statistical analyses (^31^-^33^).

Efforts to unravel the genes implicated in MASLD have been hindered by the intricacy of polygenic inheritance of those disorders which characterize MASLD – in other words, IR, obesity, and inflammation. Leptin – a pleiotropic adipokine with diverse functions – plays an essential role in obesity, IR, inflammation, and hepatic metabolism, and its levels serve as a gauge of energy stores. Leptin acts through its receptor (LEPR) in the liver and reduces the expression of the SREBP-1 gene which in turn regulates genes involved in glucose metabolism, fatty acid, and lipid production (^11^). Leptin restricts the storage of triglycerides to fat cells or lipocytes or adipocytes, and limits triglyceride storage in non-adipose organs and tissues like the liver (^34^). Further evidence corroborates the possible role of leptin in MASLD.

Leptin has a potential dual role in the initiation and progression phases of MASLD. In the initial stages of the disease, leptin has an anti-steatotic effect, but as MASLD progresses and leptin levels increase above normal levels, it exerts a proinflammatory and profibrogenic action (^34^). Other studies have shown that leptin levels appear to rise as hepatocyte steatosis develops (^7^,^12^). Leptin is also closely linked with IR. Leptin levels are positively associated with insulin (^12^) and glucose levels (^5^), as well as IR (^5^,^12^) and obesity (^5^,^13^). Under normal conditions, leptin augments insulin sensitivity through PI3K pathway in the liver, by controlling the production of hepatic glucose (^34^). With increasing fatty mass, leptin rises as a compensatory mechanism to preserve insulin sensitivity, although sustained hyperleptinemia contributes to the liver fibrogenesis (^11^).

Finally, MASLD patients usually have higher circulating leptin levels (^5^,^12^-^15^), although not always (^16^,^17^). Little is known, however, about the role of the *LEP* gene and its polymorphisms in MASLD. Interestingly, mutations that lead to leptin deficiency can result in IR and obesity (^11^).

The human *LEP* gene which contains various polymorphisms is situated at chromosomal location 7q31.3, spans approximately 18 kb, contains three exons, and encodes 167 amino acids (^21^). Since leptin and the *LEP* gene have many different functions, it is sensible to assume that *LEP* gene variants may play a role in MASLD pathogenesis. In this investigation, we detected a significant association between the -2548G>A polymorphism located in the promoter region of the *LEP* gene and MASLD. The *LEP* -2548G>A “GG” genotype compared with the ‘‘AA+AG’’ genotype had a 58% decreased risk for MASLD. The common SNP of -2548G>A might not be a marker of genetic susceptibility to MASLD by itself, instead it may co-inherit with another unkown functional marker of the *LEP* gene (linkage disequilibrium). Alternatively, because -2548G>A is a variant in the promoter region, it can alter the transcription factor binding sites and consequently affect *LEP* gene expression. In fact, this SNP is near the Sp1 transcription factor binding site, as well as two repetitive sequences of MER11 and Alu which can regulate *LEP* gene transcription (^18^,^21^). Interestingly, the rate of both adipose tissue leptin mRNA levels and leptin secretion of adipose tissue in individuals with -2548G>A “AA” genotype is twice as high as the “GA+GG” subjects. Therefore, the -2548G>A variant influences the leptin gene expression in adipocytes (^35^). The “AA” genotype also has a higher serum leptin level (^18^-^20^). More evidence is available to support the hypothesis that the “GG” genotype of *LEP* -2548G>A may have an effect on MASLD. Prior publications have reported significant associations between the *LEP* gene 2548G>A polymorphism and key risk factors for MASLD including obesity, dyslipidemia, diabetes, IR, and hypertension. Individuals with -2548G>A “AA” genotype have higher circulating levels of glucose (^21^), triglycerides (^22^), and total cholesterol (^18^), and less hepatic insulin sensitivity (^20^) against those carrying “GG” genotype. The “AA” genotype increases the risk of IR (^20^,^21^), hyperglycemia (^21^), T2DM (^22^), gestational diabetes mellitus (^21^), obesity (^23^), and hypertension (^25^,^26^) too. Moreover, the -2548 “A” allele is a risk factor for diabetes (^21^) and IR (^23^) compared to the “G” allele. Consequently, the results of our study showing the *LEP* -2548G>A “GG” genotype acts as a marker of decreased risk for MASLD are consistent with the considerations above.

There are several methodological limitations of this case-control candidate gene association study like many other studies. Due to limited funding, serum leptin level and other *LEP* gene variants besides -2548G>A were not investigated. The sample size was modest which was due to budget limitations and using liver biopsy. There are strengths that warrant attention, as well. In addition to multicenter research, this report benefits from using the gold standard method to diagnose MASLD (liver biopsy), and choosing the SNP located in the promoter region. The present study proposes a new interesting finding that is in line with earlier research.

In conclusion, to our knowledge, this is the first study that showed the “GG” genotype of *LEP* -2548G>A gene variant acted as a protective factor in susceptibility to biopsy-proven MASLD. This finding is in agreement with the hypothesis that the* LEP* gene may have a role in the pathogenesis of MASLD, although it is required to be corroborated by further studies in other populations.

## Data Availability

datasets related to this article will be available upon request to the corresponding author.
